# Urinary tract infections and antimicrobial susceptibility: A retrospective trend analysis of uropathogens in women in Accra, Ghana (2019–2022)

**DOI:** 10.1371/journal.pone.0321293

**Published:** 2025-04-04

**Authors:** Innocent Afeke, Joseph Adu-Amankwaah, Abdul-Wahab Mawuko Hamid, Precious Kwablah Kwadzokpui, Enoch Aninagyei, Glona Emmanuel, John Gameli Deku

**Affiliations:** 1 Department of Medical Laboratory Sciences, School of Allied Health Sciences, University of Health and Allied Sciences, Ho, Ghana; 2 Department of Physiology, Xuzhou Medical University, Xuzhou, China; 3 Department of Medical Laboratory, Ho Teaching Hospital, Ho, Ghana; 4 Department of Biomedical Sciences, School of Basic and Biomedical Sciences, University of Health and Allied Sciences, Ho, Ghana; North Carolina State University, UNITED STATES OF AMERICA

## Abstract

Urinary tract infections (UTIs) remain a significant public health concern, with evolving patterns in prevalence and antimicrobial resistance. This retrospective study, conducted at the Greater Accra Regional Hospital in Accra, Ghana, analyzed 11,280 urine cultures obtained exclusively from female patients from 2019 to 2022 to assess trends in UTI burden, prevalence stratified by age and month, and antimicrobial susceptibility patterns. In all, urine pathogens were isolated in 4475 (39.67%) of the samples tested. Of the total number of uropathogens isolated, majority of them were bacterial pathogens (94.21%), with an increasing proportion of fungal infections, specifically candida species (5.79%). Irrespective of the year, the highest prevalence of uropathogens were consistently recorded in the month of May, while individuals aged ≥  90 years exhibited the greatest odds of infection in 2020 (aOR: 1.88, p = 0.039). *Escherichia coli* (30.51%) and *Staphylococcus aureus* (15.16%) were the most prevalent Gram-negative and Gram-positive pathogens, respectively. Antimicrobial susceptibility testing revealed declining antibiotic effectiveness over time, with notable exceptions for gentamicin (97.4% effectiveness against *Enterococcus* spp.) and ofloxacin (82.9% against *Enterococcus* spp.). Alarmingly, most antibiotics exhibited effectiveness rates below 20% by 2022, underscoring the growing resistance challenge. These findings, drawn from a key healthcare facility in Ghana’s capital, highlight the dynamic nature of UTIs and the urgent need for targeted interventions, optimized antimicrobial stewardship, and continuous monitoring of resistance patterns to improve patient outcomes.

## Introduction

Urinary tract infections (UTIs) are among the most common infections globally, affecting individuals of all ages and placing a significant burden on healthcare systems, especially in developing countries [1,2]. UTIs are caused predominantly by uropathogens such as *Escherichia coli (E. coli)* and *Staphylococcus aureus (S. aureus)* [3,4], these infections are becoming increasingly difficult to manage due to the growing threat of antimicrobial resistance (AMR). AMR not only limits treatment options but also leads to higher morbidity, mortality, and healthcare costs [5,6].

Globally, studies have reported variations in the prevalence and etiology of UTIs across different regions, age groups, and seasons [7,8]. Research has consistently identified *E. coli* as the leading cause of community- and hospital-acquired UTIs, often followed by pathogens like *Klebsiella pneumoniae (K. pneumoniae)*, *Proteus mirabilis (P. mirabilis)*, and *Staphylococcus saprophyticus (S. saprophyticus)*. However, the susceptibility patterns of these organisms vary widely, with increasing resistance observed to commonly used antibiotics, such as fluoroquinolones, beta-lactams, and aminoglycosides [9]. In Ghana and other Sub-Saharan African countries, similar trends have been observed, though the extent and dynamics of resistance remain underexplored due to limited surveillance infrastructure [10].

Age-specific and temporal variations in UTI prevalence are also documented in the literature. Young adults, particularly females aged 20–29 years, are disproportionately affected, likely due to anatomical and behavioral factors [7,11]. Seasonal trends have also been noted, with peaks in UTI cases observed during certain months, possibly linked to climatic conditions, healthcare-seeking behavior, or antibiotic use [11,12]. Such trends underline the importance of localized studies to better understand epidemiological patterns and tailor interventions accordingly.

In Ghana, as in many parts of Sub-Saharan Africa, routine monitoring of UTI trends and antimicrobial susceptibility patterns is often limited, particularly in high-burden urban healthcare facilities. This lack of comprehensive data hampers the ability of clinicians and public health officials to address the challenges posed by these infections effectively. Furthermore, the seasonal and age-related variations in UTI prevalence and the shifting susceptibility patterns of uropathogens remain poorly understood, underscoring the need for targeted research in this area.

This study, conducted at the Greater Accra Regional Hospital in Accra, Ghana, aimed to fill this knowledge gap by analysing urine culture data from 2019 to 2022. Specifically, it sought to assess the burden and trends of UTIs, explore variations in prevalence across age groups and months, and evaluate the distribution and antimicrobial susceptibility patterns of uropathogens to provide evidence-based insights for improving clinical and public health interventions.

## Methods

### Study design and setting

This retrospective study was conducted at the Greater Accra Regional Hospital, a key healthcare facility in Ghana’s capital city. The study utilized laboratory data from urine culture tests conducted between 1^st^ January, 2019 and 31^st^ December, 2022 which was retrieved from the hospital archive data on 21^st^ August, 2023. The hospital serves a diverse urban population and is equipped with advanced diagnostic facilities, making it an ideal site for this investigation into UTIs and antimicrobial susceptibility patterns.

### Study population

The study included all urine culture samples submitted to the hospital’s microbiology laboratory from the 1^st^ January 2019 to 31st December 2022. We only included samples collected from females of all age groups (hospitalized and outpatients) who presented with clinical symptoms suggestive of UTIs, such as dysuria, urgency, or fever. We did not exclude patients with comorbidities or prior antimicrobial use.

### Data collection

Urine culture data were retrieved from the laboratory’s electronic records system. Variables extracted included patient demographics, date of sample collection, culture results, identification of uropathogens, and antimicrobial susceptibility patterns. Patients were stratified based on age. Monthly and annual distributions of culture requests and UTI prevalence were also analysed.

### Laboratory procedures

#### Sample collection and processing.

The laboratory confirmed collecting urine samples in sterile containers following standard procedures; midstream clean-catch urine was preferred for most patients, while catheterized urine samples were collected from non-ambulatory patients. Samples were processed promptly to ensure the reliability of results.

#### Urine culture for bacterial and fungal identification.

Urine samples were cultured using a calibrated loop (1 µ L or 10 µ L) to ensure accurate colony counts. Each sample was inoculated onto Cysteine Lactose Electrolyte Deficient (CLED) agar and MacConkey agar for the selective isolation of bacterial pathogens. Sabouraud Dextrose Agar (SDA) was used for the recovery of Candida species. The inoculated plates were incubated aerobically at 35–37°C for 18–24 hours, while SDA plates were incubated for up to 48 hours to allow fungal growth.

Following incubation, colonies were examined for morphological characteristics, including colour, hemolysis, and lactose fermentation. To ensure purity and suitability for further analysis, bacterial isolates were subcultured onto non-selective media such as Blood Agar (BA) or Mueller-Hinton Agar (MHA) before proceeding with identification and antimicrobial susceptibility testing. Candida isolates were subcultured on Sabouraud Dextrose Agar (SDA) to verify the purity. A purity plate was set up alongside biochemical or automated identification methods to confirm single-organism growth before susceptibility testing.

#### Bacterial identification.

Bacterial identification was conducted using the Phoenix 100 system, an automated diagnostic platform by BD Diagnostics. The system utilizes a combination of modified conventional, fluorogenic, and chromogenic substrates to detect biochemical and enzymatic activities of bacterial and fungal isolates. Urine cultures were prepared by inoculating bacterial colonies adjusted to a 0.5 McFarland standard using a nephelometer. The prepared suspension was introduced into the Phoenix identification (ID) panel, which contains 45 biochemical substrate wells and control wells, followed by system incubation and analysis.

#### Antimicrobial susceptibility testing (AST).

Antimicrobial susceptibility testing was performed using the Phoenix 100 system’s AST panel, which employs a broth microdilution method with a redox indicator for detecting bacterial growth in the presence of antibiotics. A standardized aliquot of the suspension prepared for identification was added to the AST broth, achieving a final concentration of approximately 5 × 1055 \times 10^55 × 105 CFU/mL. Antibiotic panels tested included beta-lactams, aminoglycosides, fluoroquinolones, and cephalosporins. Results were interpreted according to CLSI guidelines, with additional quality control measures implemented per the manufacturer’s recommendations.

### Outcome measures

The primary outcomes of the study were:

- The annual prevalence of UTIs during the review period.
- Monthly and age-stratified variations in UTI burden.
- The distribution of uropathogens and their antimicrobial susceptibility patterns.

### Statistical analysis

Data were analysed using SPSS software, version 27. Descriptive statistics, such as frequencies and percentages, were used to summarize categorical variables. Age and monthly distributions of UTI prevalence were assessed using chi-square tests, while trends over time were analysed using logistic regression. Adjusted odds ratios (aORs) with 95% confidence intervals (CIs) were calculated to explore factors associated with UTI prevalence. A p-value of < 0.05 was considered statistically significant.

### Ethical considerations

Ethical approval with reference number UHAS-REC A.1 [31] 23–24 was obtained from the University of Health and Allied Sciences Research Ethics Committee. In addition, written permission was sought from the management of the Greater Accra Regional Hospital for the use of data generated at the microbiology unit of the facility for the study. Our study was carried out according to the ethical standards and regulations laid down by the University of Health and Allied Sciences Research Ethics Committee. Informed consent was not sought from the participants because the study was a retrospective one. All archived data for the study was kept undisclosed and used for the study only.

## Results

### Age and monthly distribution of urine culture requests over the period under review

Over the period under review, a total of 11,280 urine culture requests were recorded, with annual proportions of 26.35%, 25.17%, 24.36%, and 24.14% for 2019, 2020, 2021, and 2023, respectively. The majority of requests were from individuals aged 20–29 years (30.36%), followed by those aged ≤ 19 years (17.58%) and 30–39 years (15.80%). Across all years, requests among the 20–29 age group remained consistently the highest, with annual proportions ranging from 23.92% to 26.26%. Monthly distribution showed the highest overall number of requests in September (9.21%), followed by December (8.98%) and January (8.69%). In 2019, the highest monthly proportion occurred in June (28.91%), whereas March showed the lowest in 2023 (19.74%). Variations were observed in monthly patterns across years, with relatively stable distributions in most age groups and months (Table 1).

**Table 1:** Age and monthly distribution of urine culture requests over the period under review.

Variables	Total N (%)	Year			
		2019 n (%)	2020 n (%)	2021 n (%)	2023 n (%)
Overall		2972 (26.35)	2839 (25.17)	2747 (24.36)	2722 (24.14)
Age group (years)					
≤ 19	1983 (17.58)	536 (27.03)	505 (25.47)	476 (24.01)	466 (23.50)
20-29	3424 (30.36)	872 (25.47)	899 (26.26)	834 (24.36)	819 (23.92)
30-39	1782 (15.80)	470 (26.38)	449 (25.20)	441 (24.75)	422 (23.69)
40-49	1250 (11.09)	334 (26.72)	297 (23.76)	298 (23.84)	321 (25.68)
50-59	855 (7.58)	227 (26.55)	203 (23.75)	215 (25.15)	210 (24.57)
60-69	705 (6.25)	196 (27.81)	170 (24.12)	172 (24.40)	167 (23.69)
70-79	731 (6.49)	200 (27.36)	177 (24.22)	179 (24.49)	175 (23.94)
80-89	360 (3.20)	94 (26.12)	93 (25.84)	90 (25.00)	83 (23.06)
≥ 90	190 (1.69)	43 (22.64)	46 (24.22)	42 (22.11)	59 (31.06)
Month					
January	980 (8.69)	258 (26.33)	251 (25.62)	234 (23.88)	237 (24.19)
February	939 (8.33)	239 (25.46)	239 (25.46)	228 (24.29)	233 (24.82)
March	917 (8.13)	251 (27.38)	234 (25.52)	251 (27.38)	181 (19.74)
April	972 (8.62)	252 (25.93)	243 (25.00)	247 (25.42)	230 (23.67)
May	942 (8.36)	247 (26.23)	234 (24.85)	234 (24.85)	227 (24.10)
June	941 (8.35)	272 (28.91)	235 (24.98)	266 (28.27)	168 (17.86)
July	879 (7.80)	222 (25.26)	216 (24.58)	222 (25.26)	219 (24.92)
August	943 (8.36)	242 (25.67)	238 (25.24)	236 (25.03)	227 (24.08)
September	1038 (9.21)	278 (26.79)	271 (26.11)	195 (18.79)	294 (28.33)
October	771 (6.84)	207 (26.85)	189 (24.52)	179 (23.22)	196 (25.43)
November	946 (8.39)	248 (26.22)	241 (25.48)	212 (22.42)	245 (25.90)
December	1012 (8.98)	256 (25.30)	248 (24.51)	243 (24.02)	265 (26.19)

### Burden and trend of UTI over the period under review

**Fig 1:**
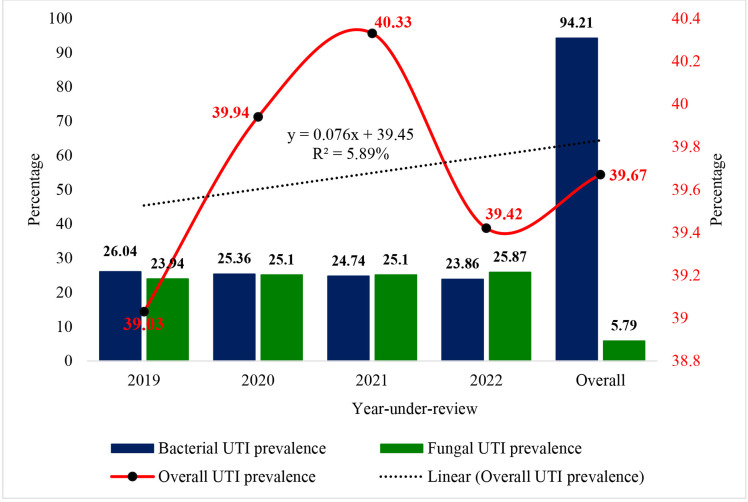
Burden and trend of UTI over the period under review.

The overall burden of UTIs was 39.67% (4475), comprising 94.21% (4216) bacterial and 5.79% (259) fungal infections. From 2019, when the prevalence was 39.03%, there was an upward trend, with prevalence increasing to 39.94% in 2020 and 40.33% in 2021. However, in 2022, the prevalence sharply declined to 39.42%. Despite this fluctuation, the overall UTI prevalence displayed an upward trajectory, with 5.59% of the variation in this trend attributed to changes during the review period. While the prevalence of bacterial and fungal UTIs remained comparable across the years, bacterial pathogens showed a consistent decline, from 26.04% in 2019 to 23.86% in 2022. In contrast, fungal UTIs exhibited an upward trend, increasing from 23.94% in 2019 to 25.87% in 2022. (Fig 1).

### Burden of UTI stratified by age and months over the period under review

In this study, the burden of UTIs showed notable variations across age groups and months, although this burden only varied significantly by age in 2019. The results showed that the highest prevalence in 2019 was observed in the 40-49 age group, 45.81% (153), while the lowest was in the 70–79 age group, 32.00% (64). Monthly, May had the highest prevalence (49.40%) and June had the lowest (29.42%). In 2020, the highest prevalence shifted to those aged ≥ 90 years of, 52.18% (24), with the lowest observed in the 60–69 age group, 35.89% (64). As in 2019, May had the highest prevalence of 53.42% (125), but October saw the lowest of 28.58% (54). In 2021, the highest prevalence occurred in the 50–59 age group, 45.12% (97), and the lowest in the 80–89 age group 32.23% (29). May continued to show the highest prevalence of 52.14% (122), while June again recorded the lowest of 28.58% (76). In 2022, the highest prevalence was again in the ≥ 90 age group, 42.38% (25), with the lowest observed in the 70–79 age group, 33.72% (59). February had the highest prevalence of 49.36% (115), while June remained the lowest of 27.98% (47) (Table 2).

**Table 2:** Burden of UTI stratified by age and months over the period under review.

Variables	2019		2020		2021		2022	
	- UTI n (%)	+ UTI n (%)	- UTI n (%)	+ UTI n (%)	- UTI n (%)	+ UTI n (%)	- UTI n (%)	+ UTI n (%)
Age group								
≤ 19	302 (56.35)	234 (43.66)	303 (60.00)	202 (40.00)	286 (60.09)	190 (39.92)	281 (60.31)	185 (39.70)
20-29	530 (60.78)	342 (39.23)	543 (60.41)	356 (39.60)	496 (59.48)	338 (40.53)	477 (58.25)	342 (41.76)
30-39	307 (65.32)	163 (34.69)	272 (60.58)	177 (39.43)	262 (59.42)	179 (40.59)	268 (63.51)	154 (36.50)
40-49	181 (54.2)	153 (45.81)	170 (57.24)	127 (42.77)	181 (60.74)	117 (39.27)	195 (60.75)	126 (39.26)
50-59	142 (62.56)	85 (37.45)	125 (61.58)	78 (38.43)	118 (54.89)	97 (45.12)	125 (59.53)	85 (40.48)
60-69	130 (66.33)	66 (33.68)	109 (64.12)	61 (35.89)	95 (55.24)	77 (44.77)	99 (59.29)	68 (40.72)
70-79	136 (68.00)	64 (32.00)	105 (59.33)	72 (40.68)	113 (63.13)	66 (36.88)	116 (66.29)	59 (33.72)
80-89	55 (58.52)	39 (41.49)	56 (60.22)	37 (39.79)	61 (67.78)	29 (32.23)	54 (65.07)	29 (34.94)
≥ 90	29 (67.45)	14 (32.56)	22 (47.83)	24 (52.18)	27 (64.29)	15 (35.72)	34 (57.63)	25 (42.38)
P-value		0.004		0.719		0.497		0.545
Month								
January	146 (56.59)	112 (43.42)	141 (56.18)	110 (43.83)	137 (58.55)	97 (41.46)	134 (56.55)	103 (43.46)
February	130 (54.4)	109 (45.61)	123 (51.47)	116 (48.54)	122 (53.51)	106 (46.50)	118 (50.65)	115 (49.36)
March	137 (54.59)	114 (45.42)	129 (55.13)	105 (44.88)	131 (52.2)	120 (47.81)	97 (53.60)	84 (46.41)
April	165 (65.48)	87 (34.53)	148 (60.91)	95 (39.10)	161 (65.19)	86 (34.82)	153 (66.53)	77 (33.48)
May	125 (50.61)	122 (49.40)	109 (46.59)	125 (53.42)	112 (47.87)	122 (52.14)	117 (51.55)	110 (48.46)
June	192 (70.59)	80 (29.42)	164 (69.79)	71 (30.22)	190 (71.43)	76 (28.58)	121 (72.03)	47 (27.98)
July	117 (52.71)	105 (47.30)	136 (62.97)	80 (37.04)	115 (51.81)	107 (48.20)	114 (52.06)	105 (47.95)
August	157 (64.88)	85 (35.13)	134 (56.31)	104 (43.70)	152 (64.41)	84 (35.60)	149 (65.64)	78 (34.37)
September	168 (60.44)	110 (39.57)	167 (61.63)	104 (38.38)	118 (60.52)	77 (39.49)	174 (59.19)	120 (40.82)
October	139 (67.15)	68 (32.86)	135 (71.43)	54 (28.58)	116 (64.81)	63 (35.20)	124 (63.27)	72 (36.74)
November	164 (66.13)	84 (33.88)	152 (63.08)	89 (36.93)	127 (59.91)	85 (40.10)	168 (68.58)	77 (31.43)
December	172 (67.19)	84 (32.82)	167 (67.34)	81 (32.67)	158 (65.03)	85 (34.98)	180 (67.93)	85 (32.08)
P-value		<0.001		<0.001		<0.001		<0.001

### Generalized linear model of predictors of UTI over the period under review

The analysis of age and monthly variations in UTI prevalence over the years 2019 to 2022, is shown in Table 3. The results showed that individuals aged 30-39 years experienced a 31% reduction in odds of UTI in 2019 (aOR of 0.69 [95% CI: 0.53 - 0.89, p = 0.004]). The 70-79 age group showed a 38% reduction in the odds of UTI in 2019 (aOR of 0.62 [0.44 - 0.88, p = 0.007]), indicating a significant protective effect. In contrast, the ≥ 90 age group had an increased odds in 2020, with odds rising by 88% (aOR of 1.88 [1.03 - 3.42, p = 0.039]). Monthly analysis revealed that April consistently demonstrated reduced odds of UTI, particularly in 2019 with a 32% reduction (aOR of 0.68 [0.48 - 0.98, p = 0.039]) and in 2022 with a 34% reduction (aOR of 0.66 [0.45 - 0.95, p = 0.027]). Conversely, June exhibited the most significant reduction across the study period, culminating in a 46% reduction in odds overall (aOR of 0.54 [0.45 - 0.66, p < 0.001]).

**Table 3:** Generalized linear model of predictors of UTI over the period under review.

Variables	2019	2020	2021	2022	Overall
	aOR [95% CI] P-value	aOR [95% CI] P-value	aOR [95% CI] P-value	aOR [95% CI] P-value	aOR [95% CI] P-value
Age group (years)					
≤ 19	1	1	1	1	1
20-29	0.84 [0.68 - 1.05] 0.136	0.97 [0.78 - 1.22] 0.801	1.02 [0.81 - 1.29] 0.850	1.12 [0.88 - 1.41] 0.353	0.98 [0.87 - 1.09] 0.684
30-39	0.69 [0.53 - 0.89] 0.004	0.97 [0.75 - 1.26] 0.816	1.03 [0.78 - 1.34] 0.845	0.89 [0.67 - 1.17] 0.387	0.88 [0.77 - 1.00] 0.051
40-49	1.09 [0.82 - 1.43] 0.557	1.11 [0.82 - 1.49] 0.505	0.96 [0.71 - 1.30] 0.805	1.00 [0.74 - 1.34] 0.993	1.04 [0.90 - 1.20] 0.629
50-59	0.78 [0.57 - 1.08] 0.132	0.93 [0.66 - 1.30] 0.668	1.25 [0.90 - 1.74] 0.182	1.08 [0.77 - 1.51] 0.672	0.99 [0.84 - 1.17] 0.886
60-69	0.66 [0.47 - 0.93] 0.018	0.83 [0.57 - 1.20] 0.318	1.25 [0.87 - 1.79] 0.222	1.08 [0.75 - 1.56] 0.686	0.92 [0.77 - 1.10] 0.346
70-79	0.62 [0.44 - 0.88] 0.007	1.05 [0.74 - 1.50] 0.767	0.90 [0.63 - 1.29] 0.559	0.79 [0.55 - 1.15] 0.224	0.82 [0.69 - 0.98] 0.029
80-89	0.95 [0.61 - 1.50] 0.835	1.04 [0.66 - 1.64] 0.866	0.72 [0.44 - 1.18] 0.194	0.87 [0.53 - 1.42] 0.586	0.89 [0.70 - 1.12] 0.322
≥ 90	0.68 [0.34 - 1.35] 0.274	1.88 [1.03 - 3.42] 0.039	0.90 [0.46 - 1.76] 0.753	1.22 [0.69 - 2.14] 0.493	1.10 [0.81 - 1.50] 0.547
Months					
January	1	1	1	1	1
February	1.10 [0.77 - 1.57] 0.615	1.20 [0.84 - 1.71] 0.328	1.24 [0.86 - 1.79] 0.251	1.27 [0.88 - 1.83] 0.198	1.19 [1.00 - 1.43] 0.055
March	1.07 [0.75 - 1.52] 0.708	1.04 [0.73 - 1.49] 0.831	1.31 [0.91 - 1.88] 0.141	1.14 [0.77 - 1.68] 0.514	1.13 [0.94 - 1.36] 0.187
April	0.68 [0.48 - 0.98] 0.039	0.82 [0.57 - 1.17] 0.276	0.76 [0.52 - 1.10] 0.142	0.66 [0.45 - 0.95] 0.027	0.73 [0.60 - 0.87] 0.001
May	1.25 [0.88 - 1.79] 0.207	1.47 [1.03 - 2.11] 0.034	1.53 [1.06 - 2.21] 0.022	1.23 [0.85 - 1.77] 0.276	1.36 [1.13 - 1.63] 0.001
June	0.54 [0.38 - 0.77] 0.001	0.55 [0.38 - 0.80] 0.002	0.57 [0.39 - 0.82] 0.003	0.51 [0.33 - 0.78] 0.002	0.54 [0.45 - 0.66] < 0.001
July	1.16 [0.80 - 1.67] 0.430	0.76 [0.52 - 1.10] 0.141	1.33 [0.92 - 1.93] 0.131	1.21 [0.84 - 1.75] 0.315	1.09 [0.91 - 1.31] 0.366
August	0.71 [0.49 - 1.02] 0.062	0.98 [0.69 - 1.41] 0.929	0.78 [0.54 - 1.13] 0.190	0.69 [0.47 - 1.00] 0.049	0.78 [0.65 - 0.94] 0.008
September	0.85 [0.60 - 1.20] 0.355	0.79 [0.56 - 1.13] 0.196	0.93 [0.63 - 1.37] 0.715	0.91 [0.64 - 1.29] 0.588	0.87 [0.73 - 1.03] 0.113
October	0.64 [0.43 - 0.93] 0.021	0.49 [0.33 - 0.74] 0.001	0.77 [0.52 - 1.16] 0.212	0.75 [0.51 - 1.11] 0.150	0.66 [0.54 - 0.80] < 0.001
November	0.67 [0.46 - 0.96] 0.030	0.75 [0.52 - 1.07] 0.114	0.96 [0.66 - 1.40] 0.831	0.59 [0.41 - 0.86] 0.006	0.72 [0.60 - 0.87] 0.001
December	0.64 [0.45 - 0.92] 0.016	0.62 [0.43 - 0.89] 0.009	0.77 [0.53 - 1.11] 0.158	0.61 [0.43 - 0.88] 0.009	0.65 [0.54 - 0.78] < 0.001

### Burden and distribution of UTI-causing bacteria and fungi over the period under review

Table 4 details the distribution of uropathogens across the study period. Of the gram-negative pathogens isolated, *E. coli* was the most prevalent overall (30.51%), consistently dominating each year followed closely by *Enterobacter spp* (15.40%). *Bacillus spp*. on the other hand was the least prevalent (0.09%) both overall and throughout the years reviewed. Among Gram-positive organisms, *Staphylococcus aureus* was the most frequently isolated (15.16%) both overall and throughout the years but with a declining trend ranging from 15.52% in 2019 to 14.54% in 2022 while *Citrobacter freundii* showed the lowest prevalence in each year, ranging from 0.09% to 0.10% (Table 4).

**Table 4:** Burden and distribution of UTI-causing bacteria and fungi over the period under review.

	Total n (%)	Year-under-review			
		2019 n (%)	2020 n (%)	2021 n (%)	2022 n (%)
Overall	4475	1160	1134	1108	1073
Organism isolated					
*Candida albicans*	259 (5.79)	62 (5.35)	65 (5.74)	65 (5.87)	67 (6.25)
Gram-positive					
*CoNS*	240 (5.37)	64 (5.52)	60 (5.30)	59 (5.33)	57 (5.32)
*Enterococcus spp*	76 (1.70)	18 (1.56)	21 (1.86)	17 (1.54)	20 (1.87)
*Staphylococcus aureus*	678 (15.16)	180 (15.52)	166 (14.64)	176 (15.89)	156 (14.54)
*Bacillus spp*	3 (0.07)	1 (0.09)	1 (0.09)	1 (0.10)	0 (0.00)
Gram-negative					
*Citrobacter freundi*	4 (0.09)	1 (0.09)	1 (0.09)	1 (0.10)	1 (0.10)
*Citrobacter spp.*	167 (3.74)	43 (3.71)	43 (3.80)	35 (3.16)	46 (4.29)
*E. coli*	1365 (30.51)	351 (30.26)	354 (31.22)	340 (30.69)	320 (29.83)
*Enterobacter spp*	689 (15.40)	181 (15.61)	177 (15.61)	164 (14.81)	167 (15.57)
*Klebsiella oxytoca*	286 (6.40)	75 (6.47)	73 (6.44)	70 (6.32)	68 (6.34)
*Klebsiella pneumoniae*	15 (0.34)	4 (0.35)	4 (0.36)	4 (0.37)	3 (0.28)
*Klebsiella spp.*	325 (7.27)	86 (7.42)	79 (6.97)	81 (7.32)	79 (7.37)
NFGNB	92 (2.06)	25 (2.16)	22 (1.95)	23 (2.08)	22 (2.06)
*P. aeruginosa*	178 (3.98)	44 (3.80)	43 (3.80)	47 (4.25)	44 (4.11)
*Proteus mirabilis*	56 (1.26)	14 (1.21)	14 (1.24)	14 (1.27)	14 (1.31)
*Proteus spp*	42 (0.94)	11 (0.95)	11 (0.98)	11 (1.00)	9 (0.84)

CoNS-Coagulase negative Staphylococcus; NFGNB-Non-lactose Fermenting Gram Negative Bacillus.

### Antimicrobial effectiveness rate and susceptibility pattern of gram-negative uropathogens

Among gram-negative uropathogens, gentamicin exhibited the highest overall effectiveness (37.4%), showing strong activity against *Klebsiella spp*. (48.9%), *Citrobacter spp* (42.5%), and *Proteus mirabilis* (57.1%). Ofloxacin (31.0%) demonstrated notable effectiveness, particularly against *Klebsiella oxytoca* (79.7%), *Proteus mirabilis* (57.1%) and *Proteus spp*. (47.6%). Among cephalosporins, cefoperazone achieved the highest susceptibility (25.6%), especially against Non-lactose Fermenting Gram Negative Bacillus (35.9%), while Piperacillin-tazobactam of the Penicillins was moderately effective (22.9%), with notable activity against *Enterobacter spp* (31.6%). Carbapenems, including ertapenem (5.0%) and meropenem (0.9%), displayed limited activity across most isolates. ciprofloxacin (21.5%) and levofloxacin (20.5%) were effective against *Klebsiella oxytoca* (59.8% vs 19.2%). and *Proteus mirabilis* (47.6% each), while tetracycline and nitrofurantoin demonstrated low overall susceptibility rates (Table 5).

**Table 5:** Antimicrobial effectiveness rate and susceptibility pattern of gram-negative uropathogens.

	Overall effectiveness rate	Gram-negative uropathogens										
		*Citrobacter freundi*	*Citrobacter spp*	*E. coli*	*Enterobacter spp*	*Klebsiella oxytoca*	*Klebsiella pneumoniae*	*Klebsiella spp*	*NFGNB*	*P. aeruginosa*	*Proteus mirabilis*	*Proteus spp*
Total uropathogens isolated	4216	4	167	1365	689	286	15	325	92	178	56	42
Antibiotics												
Cephalosporins												
1^st^ gen												
Cefazolin	39 (0.9)		4 (2.4)					3 (0.9)	4 (4.3)	4 (2.2)	8 (14.3)	4 (9.5)
2^nd^ gen												
Cefuroxime	692 (16.4)		23 (13.8)	70 (5.1)	90 (13.1)	4 (1.4)	3 (20.0)	64 (19.7)	23 (25.0)	31 (17.4)	4 (7.1)	20 (47.6)
3^rd^ gen												
Cefoperazone	1081 (25.6)	4 (100)	32 (19.2)	419 (30.7)	212 (30.8)	15 (5.2)	4 (26.7)	57 (17.5)	33 (35.9)	39 (21.9)	1 (1.8)	10 (23.8)
Ceftazidime	544 (12.9)		11 (6.6)	30 (2.2)	66 (9.6)	4 (1.4)		22 (6.8)	20 (21.7)	17 (9.6)		12 (28.6)
Ceftriaxone	715 (17.0)		22 (13.2)	72 (5.3)	74 (10.7)	4 (1.4)	3 (20.0)	64 (19.7)	23 (25.0)	31 (17.4)	4 (7.1)	20 (47.6)
Carbapenems												
Ertapenem	210 (5.0)		11 (6.6)	43 (3.2)	21 (3.0)		3 (20.0)	42 (12.9)	3 (3.3)	16 (9.0)	4 (7.1)	8 (19.0)
Meropenem	36 (0.9)		16 (1.2)	4 (0.6)			4 (1.2)					
Penicillins												
Amoxicillin/Clavulanate	209 (5.0)		11 (6.6)	42 (3.1)	21 (3.0)		3 (20.0)	42 (12.9)	3 (3.3)	16 (9.0)	4 (7.1)	8 (19.0)
Ampicillin	557 (13.2)		23 (13.8)	374 (27.4)	69 (10.0)			31 (9.5)	3 (3.3)	17 (9.6)	1 (1.8)	
Piperacillin-tazobactam	965 (22.9)	4 (100)	27 (16.2)	393 (28.8)	218 (31.6)	4 (1.4)		22 (6.8)	2 (2.2)	31 (17.4)	4 (7.1)	
Cloxacillin	39 (0.9)						3 (0.9)	4 (4.3)	4 (2.2)	8 (14.3)	4 (9.5)	
Fluoroquinolones												
Ciprofloxacin	908 (21.5)		26 (15.6)	79 (5.8)	91 (13.2)	171 (59.8)	3 (20.0)	65 (20.0)	31 (33.7)	37 (20.8)	20 (35.7)	20 (47.6)
Levofloxacin	864 (20.5)		26 (15.6)	91 (6.7)	146 (21.2)	55 (19.2)	3 (20.0)	78 (24.0)	29 (31.5)	26 (14.6)	12 (21.4)	20 (47.6)
Moxifloxacin	482 (11.4)		47 (28.1)	130 (9.5)	44 (6.4)	11 (3.8)	4 (26.7)	80 (24.6)	4 (4.3)	15 (8.4)	7 (12.5)	4 (9.5)
Nalidixic acid	7 (0.2)		7 (0.5)	7 (1.0)								
Norfloxacin	568 (13.5)		1 (0.6)	79 (5.8)	95 (13.8)	124 (43.4)		23 (7.1)	1 (1.1)	4 (2.2)	12 (21.4)	
Ofloxacin	1307 (31.0)		29 (17.4)	123 (9.0)	207 (30.0)	228 (79.7)	7 (46.7)	86 (26.5)	37 (40.2)	51 (28.7)	32 (57.1)	20 (47.6)
Macrolides												
Roxithromycin	3 (0.1)								3 (1.7)	3 (5.4)		
Aminoglycosides												
Amikacin	606 (14.4)		12 (7.2)	147 (10.8)	58 (8.4)	43 (15.0)		22 (6.8)	20 (21.7)	26 (14.6)	4 (7.1)	12 (28.6)
Gentamicin	1576 (37.4)		71 (42.5)	253 (18.5)	204 (29.6)	15 (5.2)	7 (46.7)	159 (48.9)	28 (30.4)	39 (21.9)	12 (21.4)	24 (57.1)
Tetracyclines												
Tetracycline	301 (7.1)			11 (0.8)	167 (24.2)	17 (5.9)		11 (3.4)	8 (8.7)	20 (11.2)		
Glycopeptides												
Vancomycin	515 (12.2)		54 (32.3)	142 (10.4)	44 (6.4)	11 (3.8)	4 (26.7)	83 (25.5)	4 (4.3)	16 (9.0)	7 (12.5)	4 (9.5)
Linezolid	553 (13.1)		58 (34.7)	142 (10.4)	44 (6.4)	11 (3.8)	4 (26.7)	86 (26.5)	8 (8.7)	19 (10.7)	15 (26.8)	8 (19.0)
Sulfonamides												
Co-trimoxazole	205 (4.9)	1 (25.0)		84 (6.2)	57 (8.3)			4 (1.2)		2 (1.1)		1 (2.4)
Others												
Chloramphenicol	490 (11.6)		15 (9.0)	29 (2.1)	66 (9.6)	4 (1.4)		25 (7.7)	24 (26.1)	19 (10.7)	8 (14.3)	16 (38.1)
Daptomycin	485 (11.5)		47 (28.1)	130 (9.5)	44 (6.4)	11 (3.8)	4 (26.7)	83 (25.5)	4 (4.3)	15 (8.4)	7 (12.5)	4 (9.5)
Lincomycin	68 (1.6)		26 (1.9)	11 (1.6)			12 (3.7)		4 (2.2)	1 (1.8)		
Mupirocin	505 (12.0)		50 (29.9)	142 (10.4)	44 (6.4)	11 (3.8)	4 (26.7)	80 (24.6)	4 (4.3)	15 (8.4)	7 (12.5)	4 (9.5)
Nitrofurantoin	105 (2.5)					39 (13.6)					4 (7.1)	

NFGNB-Non-lactose Fermenting Gram Negative Bacillus.

### Antimicrobial effectiveness rate and susceptibility pattern of gram-positive uropathogens

Among the cephalosporins, cefoperazone exhibited a substantial effectiveness rate of 52.5% for CoNS, while ceftazidime demonstrated high effectiveness against *Staphylococcus aureus* (77.6%) and ceftriaxone showed even higher effectiveness (85.5%) in the same organism. Furthermore, piperacillin-tazobactam had a notable effectiveness of 58.3% for CoNS and 14.3% for *Staphylococcus aureus*. In the fluoroquinolone category, levofloxacin displayed an impressive effectiveness of 69.7% against *Enterococcus spp*, while ofloxacin yielded the highest overall effectiveness rate of 82.9% against *Enterococcus spp*. In the aminoglycoside group, gentamicin was particularly effective against *Enterococcus spp* (97.4%) and 68.1% for *Staphylococcus aureus*. *Bacillus spp*. was 100% susceptible to moxifloxacin, gentamicin, vancomycin, linezolid, daptomycin, and mupirocin (Table 6).

**Table 6:** Antimicrobial effectiveness rate and susceptibility pattern of gram-positive uropathogens.

	Overall effectiveness rate	Gram-positive			
		CoNS	Enterococcus spp	Staphylococcus aureus	Bacillus spp
Total uropathogens isolated	4216	240	76	678	3
Antibiotics					
Cephalosporins					
1^st^ generation					
Cefazolin	39 (0.9)			4 (0.6)	
2^nd^ generation					
Cefuroxime	692 (16.4)	40 (16.7)	1 (1.3)	220 (32.4)	
3^rd^ generation					
Cefoperazone	1081 (25.6)	126 (52.5)		112 (16.5)	
Ceftazidime	544 (12.9)	28 (11.7)	59 (77.6)	195 (28.8)	
Ceftriaxone	715 (17)	40 (16.7)	65 (85.5)	195 (28.8)	
Carbapenems					
Ertapenem	210 (5)	12 (5.0)		29 (4.3)	
Meropenem	36 (0.9)		4 (0.6)		
Penicillins					
Amoxicillin/clavulanate	209 (5)	12 (5.0)		29 (4.3)	
Ampicillin	557 (13.2)	31 (12.9)		6 (0.9)	
Piperacillin-tazobactam	965 (22.9)	140 (58.3)		97 (14.3)	
Cloxacillin	39 (0.9)		4 (0.6)		
Fluoroquinolones					
Ciprofloxacin	908 (21.5)	40 (16.7)	9 (11.8)	199 (29.4)	
Levofloxacin	864 (20.5)	16 (6.7)	53 (69.7)	207 (30.5)	
Moxifloxacin	482 (11.4)	18 (7.5)		48 (7.1)	3 (100)
Nalidixic acid	7 (0.2)				
Norfloxacin	568 (13.5)	1 (0.4)	8 (10.5)	208 (30.7)	
Ofloxacin	1307 (31)	47 (19.6)	63 (82.9)	252 (37.2)	
Macrolides					
Roxithromycin	3 (0.1)				
Aminoglycosides					
Amikacin	606 (14.4)	7 (2.9)	1 (1.3)	174 (25.7)	
Gentamicin	1576 (37.4)	59 (24.6)	74 (97.4)	462 (68.1)	3 (100)
Tetracyclines					
Tetracycline	301 (7.1)	3 (1.3)		54 (8.0)	
Glycopeptides					
Vancomycin	515 (12.2)	18 (7.5)		56 (8.3)	3 (100)
Linezolid	553 (13.1)	18 (7.5)		60 (8.8)	3 (100)
Sulfonamides					
Co-trimoxazole	205 (4.9)	33 (13.8)		23 (3.4)	
Others					
Chloramphenicol	490 (11.6)	28 (11.7)	1 (1.3)	168 (24.8)	
Daptomycin	485 (11.5)	18 (7.5)		48 (7.1)	3 (100)
Lincomycin	68 (1.6)		2 (0.3)		
Mupirocin	505 (12)	18 (7.5)		56 (8.3)	3 (100)
Nitrofurantoin	105 (2.5)	3 (1.3)	58 (76.3)		

CoNS-Coagulase negative Staphylococcus.

### Trend in antibiotic susceptibility pattern over the period under review

**Fig 2:**
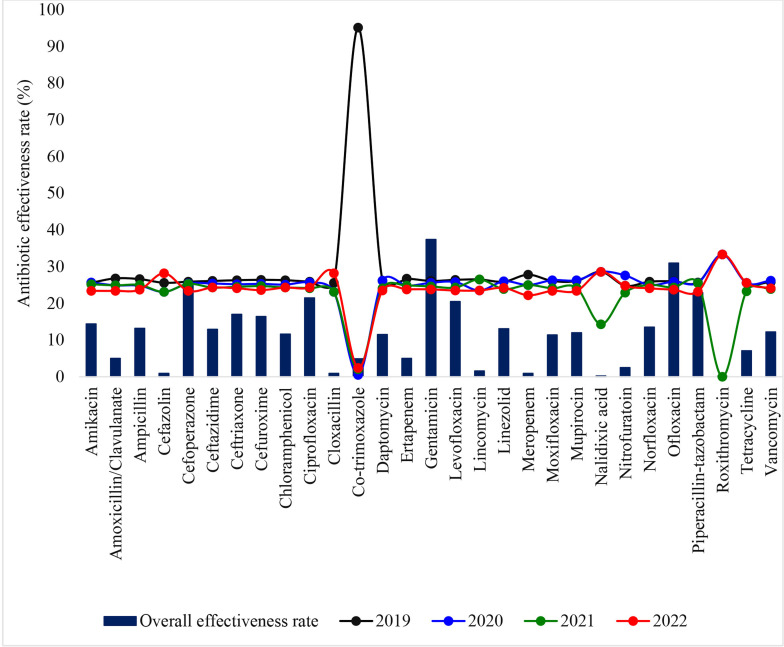
Trend in antibiotic susceptibility pattern over the period under review.

As depicted in Fig 2, most of the antibiotics except for cefoperazone, ciprofloxacin, gentamicin, ofloxacin, and piperacillin-tazobactam had overall effectiveness rates below 20%. The results reveal that generally, antibiotic effectiveness rate reduced with advancement in years.

## Discussion

This study investigated the prevalence of urinary tract infections (UTIs), the distribution of uropathogens, and their antimicrobial susceptibility patterns among Greater Accra Regional Hospital patients. The annual UTI prevalence was 39.67%, with significant age and seasonal variations. *E. coli* was the predominant uropathogen, followed by *K. pneumoniae* and *Pseudomonas aeruginosa*. The antimicrobial susceptibility testing revealed high resistance rates to commonly prescribed antibiotics, particularly ampicillin and ciprofloxacin, while nitrofurantoin and meropenem demonstrated lower resistance rates. Our findings align with global and regional trends where *E. coli* is consistently the most prevalent uropathogen [3,13,14]. Studies from Ghana and other sub-Saharan African countries, have similarly reported *E. coli* dominance, attributing this to its virulence factors, including fimbriae and adhesins that facilitate urinary tract colonization [10,15]. The high resistance rates observed for ciprofloxacin and ampicillin are consistent with studies from Ghana [16] and Romania [17], highlighting the widespread misuse of antibiotics and the lack of robust antimicrobial stewardship programs in low-resource settings. In contrast, the lower resistance rates to nitrofurantoin and meropenem align with findings from Saudi Arabia [18], potentially due to their less frequent use in empirical therapy, preserving their efficacy. However, our study contrasts with findings from high-income countries, such as the United States and Europe, where resistance to ciprofloxacin is less pronounced [19,20]. This difference may be explained by stricter regulatory frameworks and more effective surveillance systems in those regions. The relatively lower prevalence of multidrug-resistant *K. pneumoniae* in our study compared to reports from tertiary hospitals in Egypt [21] could reflect differences in healthcare settings. The Greater Accra Regional Hospital primarily caters to a non-tertiary patient population, which might account for the reduced exposure to antibiotics typically associated with resistance selection. Conversely, the higher resistance rates to first-line antibiotics in this study compared to a similar study conducted in Volta Region, Ghana [10], might stem from differences in prescription practices or patient populations. Urban settings like Accra often face higher antimicrobial resistance due to the over-the-counter availability of antibiotics.

Our study underscores the pressing need for targeted interventions to curb UTI prevalence and antimicrobial resistance. The findings suggest that nitrofurantoin and meropenem should be prioritized for empirical treatment of UTIs in this population. However, the high resistance rates to commonly used antibiotics highlight the urgency of implementing antimicrobial stewardship programs and public health education campaigns to mitigate inappropriate antibiotic use. Furthermore, the significant age variations in UTI prevalence observed in our study emphasize the importance of tailoring interventions to specific demographic groups thus women in their 40-59 and those over 90 years. The variations in UTI prevalence across different age groups observed in this study can be attributed to physiological, hormonal, and lifestyle factors, as well as disruptions in healthcare access due to the COVID-19 pandemic. The highest UTI prevalence in 2019 and 2021 was recorded in women aged 40–59 years. This trend is likely influenced by the hormonal changes associated with perimenopause and menopause, where declining estrogen levels lead to thinning of the vaginal and urethral epithelium. These changes reduce the natural defense mechanisms against bacterial infections, making women in this age group more susceptible to UTIs. Additionally, women in their 40s and 50s often maintain active sexual lives, and sexual activity is a well-documented risk factor for UTIs due to bacterial transfer to the urethra [22]. The COVID-19 pandemic further increased the risk of UTIs in this age group by disrupting routine healthcare services, including preventive screenings and the management of conditions like diabetes and urinary tract abnormalities. Since underlying health conditions are known to predispose individuals to UTIs [23], the reduced access to healthcare during the pandemic likely contributed to the higher prevalence observed in these years.

In both 2020 and 2022, the highest prevalence of UTIs was observed among women aged ≥ 90 years. This increase can be attributed to several age-related factors, such as a decline in immune function, incontinence, and reduced mobility. These factors contribute to poor perineal hygiene, which in turn raises the risk of recurrent infections. This finding aligns with similar studies [24]. Additionally, the COVID-19 pandemic likely worsened the situation. During the pandemic’s peak, healthcare systems were overwhelmed, leading to delays in routine medical care, including UTI diagnosis and treatment. Elderly women, especially those aged ≥ 90 years, may have struggled to access healthcare due to movement restrictions and limited caregiver support, making them more susceptible to infections. Their challenges with incontinence and hygiene further increased the risk. The lack of timely intervention and follow-up care may have resulted in prolonged or more frequent UTI episodes, as observed in a study from Spain [25].

UTI prevalence also varied significantly between the dry and wet seasons, the two major seasons in Ghana. The highest prevalence was consistently recorded in May, during the peak of the wet season, when increased humidity and changes in hygiene practices may favour bacterial growth and UTI development. Conversely, June and October recorded lower prevalence rates, potentially due to improved hydration and cooler temperatures, which may reduce bacterial proliferation and urinary retention. Consistent with our study, researchers have also observed seasonal variations in UTI prevalence [26,27]. During the dry season (November–March), especially in February, higher UTI rates were noted. This pattern may be linked to dehydration and more concentrated urine, which create conditions that favor bacterial growth. Moreover, limited access to clean water during the dry season could negatively affect hygiene practices, further raising the risk of UTIs [12,27,28].

One strength of this study is its large dataset, which enhances the reliability of the findings. Additionally, the use of automated bacterial identification and antimicrobial susceptibility testing ensures accuracy and reproducibility. However, the study has limitations, including its retrospective design, which precludes the assessment of clinical outcomes. Furthermore, as the study was conducted in a single center, the findings may not be generalizable to other regions in Ghana. Our study highlights the urgent need for national and institutional-level surveillance systems to monitor antimicrobial resistance trends. Further research involving multicenter studies and incorporating molecular analyses to understand resistance mechanisms is recommended. In addition, policymakers should enforce stricter regulations on antibiotic sales and usage to curb the rising threat of resistance.

This study has several limitations that should be considered when interpreting the findings. First, its retrospective design precluded the assessment of clinical outcomes, such as treatment success or recurrence rates, which could provide a more comprehensive understanding of the clinical impact of antimicrobial resistance. Second, as the study was conducted at a single urban hospital, the findings may not be generalizable to other regions of Ghana, particularly rural areas, where access to healthcare and antibiotic usage patterns may differ. Third, the absence of molecular analyses to characterize resistance mechanisms limits our ability to identify the genetic factors driving antimicrobial resistance among the uropathogens. Variations in sample collection and diagnostic practices over the study period could have introduced bias in estimating prevalence and resistance trends. Furthermore, we were unable to access detailed clinical observations that justified the request for urine cultures. This limits our ability to correlate microbiological findings with specific clinical presentations and outcomes. Moreover, missing information on pregnancy status and whether hospital admissions were related to pregnancy complications, including undetected UTIs, restricted our ability to assess potential risk factors for infection. Finally, we could not distinguish between hospital-acquired and community-acquired infections among inpatients, which limits the contextual interpretation of antimicrobial resistance patterns and their implications for infection control strategies.

## Conclusion

This study underscores the significant burden of UTIs and the alarming antimicrobial resistance trends at the Greater Accra Regional Hospital in Ghana. With *E. coli* as the predominant uropathogen and high resistance rates to commonly used antibiotics like ampicillin and ciprofloxacin, the findings highlight the urgent need for antimicrobial stewardship programs and stricter antibiotic regulation. Lower resistance rates to nitrofurantoin and meropenem suggest their suitability for empirical therapy in this population. The study emphasizes the importance of establishing robust national surveillance systems that capture detailed data on antimicrobial resistance patterns, uropathogen prevalence, and antibiotic usage across various regions. Such surveillance would enable timely identification of emerging resistance trends and inform targeted interventions. Additionally, focused public health campaigns aimed at combating antibiotic resistance are crucial, particularly in promoting proper antibiotic use, infection prevention, and awareness among healthcare workers and the general public. These efforts should be aligned with national strategies for antimicrobial stewardship. Future multicenter studies and molecular analyses are essential for a deeper understanding of resistance mechanisms, which will guide the development of effective and targeted interventions.

**Importance of the Study**

- Guides Clinical Practice: The study provides critical insights into the prevalence and antimicrobial susceptibility patterns of uropathogens, helping clinicians make evidence-based decisions for the treatment of UTIs in Ghana.
- Informs Public Health Strategies: By highlighting age and seasonal variations in UTI prevalence, the findings enable targeted public health interventions to address high-risk groups and periods.
- Monitors Antimicrobial Resistance: The declining effectiveness of commonly used antibiotics emphasizes the need for enhanced antimicrobial stewardship programs and the development of alternative treatment strategies.
- Improves Regional Healthcare Policy: The study, conducted in a key healthcare facility in Ghana’s capital, offers valuable data to policymakers for improving diagnostic and therapeutic protocols in regional hospitals across the country.
